# Schulische Alkoholprävention mittels Virtual Reality

**DOI:** 10.1007/s00103-022-03541-y

**Published:** 2022-05-13

**Authors:** Christiane Stock, Christina Prediger, Robert Hrynyschyn, Stefanie Helmer

**Affiliations:** Institut für Gesundheits- und Pflegewissenschaft, Charité – Universitätsmedizin Berlin, Corporate member of Freie Universität Berlin and Humboldt-Universität zu Berlin, Augustenburger Platz 1, 13353 Berlin, Deutschland

**Keywords:** Alkoholprävention, Jugendliche, Virtuelle Simulation, E-Health Anwendung, Gruppendruck, Alcohol prevention, Adolescents, Virtual simulation, E-health application, Peer pressure

## Abstract

Riskanter Alkoholkonsum ist bei Jugendlichen in Deutschland nach wie vor von hoher Public-Health-Relevanz, weshalb vorbeugend die Kompetenzen von Jugendlichen im Umgang mit Alkohol und Gruppendruck gefördert werden sollten. Interaktive und geschlechtssensible schulische Primärpräventionsangebote besitzen ein großes Potenzial, die Erreichbarkeit der Zielgruppe und die Effektivität von Interventionen zu fördern. Dabei können virtuelle Simulationen als digitales Medium genutzt werden.

Virtual Reality (VR) ermöglicht die Erfahrung von risikobehafteten Situationen in sicherer Umgebung. International gibt es zwei Alkoholpräventionsprojekte für Jugendliche, die VR einsetzen. Die gemeinsame Entwicklung mit der Adressat*innengruppe war dabei ein bedeutendes Kernelement und es wurden bereits umfassende Untersuchungen zur Benutzungsfreundlichkeit sowie zur subjektiven Wirksamkeit durchgeführt. Gängige Effektivitätsevaluationen wie randomisierte kontrollierte Studien kommen bei interaktiven Formaten allerdings an ihre Grenzen, weshalb auch alternative und ergänzende Evaluationsansätze zukünftig eine Rolle spielen sollten. Zusätzlich muss untersucht werden, inwiefern VR-Simulationen auf Rezipient*innen zugeschnitten werden können. Hierbei ist die gendersensible Gestaltung gleichzeitig als Potenzial und als Herausforderung zu sehen.

Auch in Deutschland sollte die Möglichkeit des Einsatzes von VR in der Alkoholprävention bei Jugendlichen vertiefend untersucht werden.

## Abstract (en)

Risky alcohol consumption among adolescents continues to be of high public health relevance in Germany. Therefore, preventive measures should promote the skills of adolescents to deal with alcohol and peer pressure. Interactive and gender-sensitive primary prevention programs in schools have a great potential to reach the target group and to increase the effectiveness of interventions. Digital tools such as virtual simulations may help with this process.

Virtual reality (VR) enables the experience of risk situations in a safe environment. Internationally, there are currently two VR alcohol prevention programs for adolescents. Their co-creation with the target group was a core element, and comprehensive research on the usability and subjective effectiveness has already been published. Conventional effectiveness evaluations such as randomized controlled trials, however, have limitations with interactive formats. Alternative and complementary evaluation approaches are needed in the future. Furthermore, the potential of tailoring VR to recipients has to be examined. In this context, a gender-sensitive design is both an opportunity and a challenge.

Further research is needed to study possibilities to use VR in alcohol prevention in adolescents in Germany.

## Einleitung

Die Prävalenz des Alkoholkonsums und insbesondere von riskantem Konsum, wie z. B. Rauschtrinken, ist weiterhin hoch, auch wenn laut aktuellen Daten der Alkoholkonsum unter Jugendlichen in Europa in den letzten Jahren leicht, aber stetig zurückgegangen ist [1]. So gaben im European School Survey Project for Alcohol and other Drugs von 2019 (ESPAD) 90 % der 15- und 16-jährigen Schüler*innen in Deutschland an, mindestens einmal in ihrem Leben Alkohol konsumiert zu haben [1]. 65 % berichteten, in den letzten 30 Tagen Alkohol konsumiert zu haben, und 20 % hatten in den letzten 30 Tagen mindestens einmal 5 oder mehr alkoholische Getränke bei einem Anlass konsumiert (Rauschtrinken; [1]). Die Drogenaffinitätsstudie der Bundeszentrale für gesundheitliche Aufklärung (BZgA) 2019 weist für 12- bis 17-Jährige in Deutschland eine Alkoholkonsum-Lebenszeitprävalenz von 63 % aus, in Bezug auf Rauschtrinken liegt diese bei 15 % [2].

Im Hinblick auf Geschlechtsunterschiede zeigen die ESPAD-Daten für Deutschland eine höhere 30-Tages-Prävalenz unter Mädchen gegenüber Jungen (68 % vs. 63 %), jedoch geben Jungen geringfügig häufiger als Mädchen an, betrunken gewesen zu sein (21 % vs. 19 %). Dies deckt sich mit den Daten der Drogenaffinitätsstudie, welche unter Jungen eine weitere Verbreitung von Rauschtrinken aufweist als unter Mädchen [3]. Zudem gibt es Unterschiede, je nachdem welcher Schultyp besucht wird: So zeigte sich bei Jugendlichen an Gymnasien eine signifikant niedrigere Prävalenz des regelmäßigen und des riskanten Alkoholkonsums als bei Jugendlichen, die andere Schultypen besuchen [4].

Insgesamt weisen die Daten zum Alkoholkonsum unter Schüler*innen in Deutschland einen Handlungsbedarf auf, effektive präventive Ansätze in diesem Bereich zu stärken und weiterzuentwickeln sowie diese an spezifische Bedürfnisse der Adressat*innengruppe anzupassen. Solch eine Primärprävention bei Jugendlichen ist entscheidend, da Alkoholkonsum ein wesentlicher Risikofaktor für eine Reihe von gesundheitlichen, sozialen und möglichen rechtlichen Konsequenzen ist [5]. So ist ein früher Alkoholkonsumbeginn assoziiert mit einer höheren Wahrscheinlichkeit für Alkoholmissbrauch im späteren Leben sowie der Entwicklung von Alkoholabhängigkeit [6–8]. Des Weiteren ist Alkoholkonsum in der Jugend mit unerwünschten Schwangerschaften, Kriminalität und Schulversagen verbunden [5].

Präventionsmaßnahmen kommen bei Jungen und Mädchen unterschiedlich an und variieren in ihrer Wirkung [9]. Wenn Jungen erreicht werden, zeigten sich bei Programmen zunächst teilweise stärkere, langfristig aber wieder nachlassende Effekte in der Alkoholreduktion als bei Mädchen [10]. Für Jungen sind interaktive Komponenten bei Präventionsangeboten besonders wichtig, während für Mädchen die Vermittlung von verhaltensbezogenen Lebenskompetenzen bedeutender sind [10]. Insgesamt sind Ansprache, Einbindung und Anpassung von Präventionsprogrammen an die Bedürfnisse von Jugendlichen zentrale Aspekte für wirksame Präventionsprogramme [11].

Ein besseres Verständnis dessen, was Jugendliche dazu veranlasst, Alkohol zu konsumieren, hat zu optimierten Präventionsprogrammen geführt. Als einer der bedeutendsten Gründe für den Beginn und die Beibehaltung des Alkoholkonsums gilt dabei Gruppendruck unter Jugendlichen [12–14]. Obwohl diverse, an sozialen Einflüssen einsetzende Präventionsprogramme in ihrer Wirkung keine einheitlichen Ergebnisse zeigen, werden solche Ansätze in Reviews hingegen insgesamt positiv bewertet [15]. Die Mehrzahl der als effektiv bewerteten Präventionsprogramme, die im deutschen Schulsetting durchgeführt und evaluiert wurden, umfassen Aspekte eines Kompetenztrainings und adressieren u. a. die Fertigkeiten, Gruppenzwang zu widerstehen (z. B. Aktion Glasklar, ALF, IPSY, Klasse 2000, Unplugged (s. Kurzbeschreibungen auf grüne-liste-praevention.de)).

Dieser Artikel stellt dar, inwieweit schulische Alkoholprävention Virtual Reality (VR) nutzen kann, um die Kompetenzen von Schüler*innen im Umgang mit Alkohol zu stärken. Dabei werden ein Überblick über die bisherigen Forschungs- und Entwicklungsprojekte und Evaluationsergebnisse in diesem Bereich gegeben, auf Möglichkeiten der zugeschnittenen Konzipierung von Interventionen nach Geschlecht eingegangen und die Chancen und Grenzen dieser Ansätze für die schulische Alkoholprävention herausgearbeitet.

## Alkoholprävention bei Jugendlichen und Möglichkeiten durch Virtual Reality

Die Adoleszenz, von der Weltgesundheitsorganisation (WHO) als Altersspanne zwischen 10 und 19 Jahren definiert [16], ist die Lebensphase, welche am meisten durch Veränderungen, Transitions- und Entwicklungsprozesse geprägt ist. Diese Veränderungen und Prozesse bringen für die Jugendlichen Herausforderungen mit sich, denen in angemessener Weise begegnet werden muss, um gesund zu bleiben [17]. Wichtig ist auch die Entwicklung von Fertigkeiten, schädlichem Verhalten gegenüber resilient zu sein. An diesem Punkt setzen Präventionsprogramme zur Kompetenz- und Resilienzsteigerung an. Eine gute Möglichkeit sind sogenannte Life-Skills-Programme, die Jugendliche dazu befähigen, eigene Entscheidungen für ein gesundes Leben zu treffen [18].

Weiterhin ist es in dieser Lebensphase wichtig, den sozialen Druck, dem Jugendliche in ihrem Umfeld ausgesetzt sein können, im Rahmen von Präventionsmaßnahmen zu adressieren. So können Jugendliche beispielsweise im Sinne der sozialen Inokulationstheorie [19] auf kritische Situationen vorbereitet werden, Vorurteile bereits frühzeitig kennenlernen und Gegenargumente entwickeln, um eine eigene Entscheidung treffen zu können [20]. Traditionell werden Trainings zur Stärkung von sozialen Kompetenzen, Widerstandsressourcen und Resilienz mit Rollenspielen (z. B. in Schulen) durchgeführt. Diese Trainings sind sowohl zeitintensiv als auch teuer in der Durchführung, da die Durchführenden spezifisch geschult werden müssen. Daher könnten virtuelle Simulationen Vorteile gegenüber dem Rollenspiel in Präsenz bieten. Dies sollte in Kosten-Nutzen-Analysen weitergehend untersucht werden.

VR, definiert als digitale 3‑D-simulierte Erfahrung [21], welche mithilfe spezieller Soft- und Hardware erlebbar gemacht wird, gilt aufgrund der erweiterten Sinneseinbindung als innovatives Medium und wurde bereits in der Gesundheitsförderung und -prävention eingesetzt (z. B. zur Prävention von Stress [22] oder Substanzkonsum [23]). Die bisher verwendeten Einsatzmöglichkeiten von VR lassen sich anhand von 3 zentralen und eng miteinander verknüpften Dimensionen unterscheiden: Präsenz, Immersion und Interaktivität [24]. Während Präsenz sich auf das subjektive Erfahren einer Umgebung bezieht, stellt die Immersion die subjektive Einschätzung der Nutzenden (z. B. das Gefühl, in der virtuellen Welt „gefangen und absorbiert“ zu sein) und die Konfiguration des VR-Systems dar. Die Interaktivität von VR drückt sich dadurch aus, dass Nutzende Einfluss auf die virtuelle Umwelt ausüben können [25]. Durch diese 3 Dimensionen ermöglicht VR das Erleben von bestimmten Situationen in einer virtuellen Umgebung [26] und kann das Lernen durch ein Gefühl der Präsenz und eine erhöhte Interaktivität im Vergleich zu traditionellen Lernumgebungen stimulieren [27].

Die zuvor beschriebenen Eigenschaften von VR lassen sich gut auf das Anwendungsfeld der Alkoholprävention übertragen, da gegebenenfalls risikobehaftete Situationen (z. B. eine Party [26]) in einer sicheren, virtuellen Umgebung erlebt werden können [28].

Darüber hinaus bietet VR durch die Auswahl von Spielcharakteren (Avataren), z. B. nach Geschlecht, die Möglichkeit, die Identifikation mit Spielfiguren zu erhöhen sowie den Simulationsverlauf dem Avatar entsprechend anzupassen. Da auf Rezipient*innen zugeschnittene Interventionen wirkungsvoller sind [29, 30], ist VR ein vielversprechendes Medium für die Alkoholprävention.

## Stand der Forschung und Entwicklung

Bisher gibt es nur wenige virtuelle Simulationen in der Alkoholprävention bei Jugendlichen, die wissenschaftlich entwickelt und evaluiert wurden [31]. Diese beziehen sich auf Jugendliche zwischen 12 und 16 Jahren. Erste Ansätze wurden in Australien (*VR House Party*, Griffith University, Brisbane [32, 33]) und Dänemark (*VR FestLab*, University of Southern Denmark, Odense [34]) entwickelt. Beide VR-Simulationen sind als Kompetenztraining zum Umgang mit Alkohol und sozialem Druck aufgebaut. So können sich die Spielenden durch eine simulierte Party bewegen und verschiedene Entscheidungen in Situationen treffen, in denen sie von Peers entweder zum Trinken von Alkohol oder von nichtalkoholischen Getränken sowie zu verschiedenen Aktivitäten (Tanzen, Spiele, Flirten) animiert werden. Dies soll das Bewusstsein für die Beeinflussung eigener Entscheidungen durch sozialen Druck erhöhen sowie verschiedene Handlungs- und Kommunikationsstrategien im Umgang mit Alkohol und Gruppendruck trainieren. Bisherige Publikationen beschreiben insbesondere die partizipativen Entwicklungsprozesse mit den Adressat*innen [32, 34–36]. Das in Zusammenarbeit mit der Adressat*innengruppe („Co-Creation-Prozess“) entstandene *VR-FestLab*-Projekt zeigte, dass ein partizipativer Ansatz entscheidend ist, um effektive VR-Gesundheitsförderungsmaßnahmen zu entwickeln [35]. Durch die Nutzung von explizitem und implizitem Wissen mehrerer Stakeholder, inklusive Personen aus der Adressat*innengruppe, und eine dadurch hervorgerufene kollaborative Wissensgenerierung können realistische VR-Anwendungsszenarien geschaffen werden [37]. Überdies kann die Offenheit und Zusammenarbeit in partizipativen Prozessen das Selbstvertrauen der Einzelnen stärken, indem sie das Gefühl des Vertrauens in die Leistung und den Beitrag zur Entwicklung der gewünschten Intervention fördern [37].

Laut ersten Evaluationen wurde das australische VR-Simulationsspiel *VR House Party* von Jugendlichen insgesamt mit hoher Akzeptanz sowie als attraktiv und zufriedenstellend bewertet [33]. Auch das dänische *VR FestLab* wurde in einer Pilottestung bezüglich Benutzungsfreundlichkeit und Spielerfahrung positiv bewertet [38]. Hierzu setzten die Forscher*innen in einer Mixed-Methods-Studie den User Experience Questionnaire (UEQ) ein und führten qualitative Interviews mit den Nutzenden durch. Das VR-Simulationsspiel wurde laut UEQ insbesondere in den Kategorien Einfachheit, Klarheit und Ansprechbarkeit hoch bewertet. In den qualitativen Interviews betonten die Nutzenden die Stärken von VR in der Alkoholprävention [38]. Jugendliche ohne Alkoholerfahrung lobten die Möglichkeit, eine Party erleben zu können, ohne sie aktiv besuchen zu müssen. Jugendliche mit Alkoholerfahrung hingegen empfanden das VR-Simulationsspiel als realistisch und hatten den Eindruck, auf einer realen Party zu sein und positive wie auch negative Partyerfahrungen sammeln zu können [38].

Eine clusterrandomisierte kontrollierte Studie, welche die Wirksamkeit der Anwendung *VR FestLab* im Hinblick auf eine erhöhte Kompetenz im Umgang mit Alkohol und Gruppendruck untersucht hat, konnte jedoch keine signifikante Erhöhung der Selbstwirksamkeitserwartung von Jugendlichen zeigen, Alkohol abzulehnen [39]. Die Gründe könnten entweder im Studiendesign (z. B. zu geringe Stichprobengröße und/oder zu kurze Spieldauer) oder aber in Mängeln der edukativen Elemente der Applikation liegen, sodass weitere Forschung zur Wirksamkeit notwendig ist. Es ist zukünftig auch wichtig, verlässliche Bewertungsmethoden anzuwenden, um die Wirksamkeit und Effektivität von Gesundheitsförderungsmaßnahmen, wie z. B. einem virtuellen Stimulationsspiel, zu messen. Randomisierte kontrollierte Studien (RCT) gelten als das primäre Forschungsdesign für den Nachweis kausaler Beziehungen zwischen Intervention und Outcome [40]. Durch die Randomisierung und die strenge Kontrolle des Umfelds können in RCT Selektionsverzerrungen und Störfaktoren minimiert werden. Mit dem verstärkten Einzug von innovativen und interaktiven Medien in die Gesundheitsförderung und Prävention werden jedoch auch die Herausforderungen in der Evaluation durch RCT deutlich: Insbesondere die lange Studiendauer von RCT wird als Kernproblem bei der Evaluation von digitalen Gesundheitsförderungsmaßnahmen gesehen, da die Technologie nach Abschluss der Wirksamkeitsmessung längst veraltet sein kann [41]. Diese Limitation trifft auch auf VR zu, da diese Technologie stetig weiterentwickelt wird und nur ein auf aktuellen technischen Standards beruhendes Spiel eine hohe Zufriedenheit bei Nutzenden erreichen wird.

Daraus sollte nicht der Schluss gezogen werden, dass die Verwendung von RCT *per se* falsch für die Bewertung von Interventionen im Bereich der virtuellen Simulationen ist. Es sollten aber alternative oder ergänzende Bewertungsansätze in Betracht gezogen werden, um verschiedene Forschungsfragen bei der Entwicklung, Anpassung und Erprobung zu beantworten. Hierzu hat das britische Medical Research Council bereits einige Vorschläge unterbreitet, die jedoch bisher nur vereinzelt angewendet werden (z. B. Stepped-Wedge-Designs, Preference Trials oder N‑of‑1 Trials; [42]).

## Genderaspekte in der virtuellen Simulation

Geschlecht als eine bedeutende und komplexe soziale Determinante von Gesundheit [43] stellt eine mögliche Kategorie dar, um Interventionen adressat*innengerecht zuzuschneiden. Ausschreibungen des Bundesministeriums für Gesundheit (BMG) im Jahr 2020 und der BZgA 2019 zu geschlechtsspezifischen Ansätzen in Prävention und Gesundheitsförderung unterstreichen den Forschungsbedarf. Da Unterschiede zwischen den Geschlechtern hinsichtlich des Konsums und des sozialen Umgangs mit Alkohol bestehen [1, 4] und diese ebenfalls die Wirksamkeit von Prävention und Gesundheitsförderung beeinflussen [44, 45], scheint es von großer Bedeutung zu sein, das Potenzial der VR-basierten Alkoholprävention geschlechtsspezifisch zu nutzen.

Die Forderung nach geschlechtsspezifischen Interventionen könnte zunächst die Konzeption einer männlichen und einer weiblichen Simulationsvariante nahelegen. Unter Berücksichtigung gelebter Vielfalt, rechtlicher Entwicklungen (beispielsweise das veränderte Personenstandsgesetz seit 2018) und gendertheoretischer Ansätze greift eine solche binäre Differenzierung jedoch zu kurz. So können Interventionen, die nach Mädchen und Jungen differenzieren, Stereotypen reproduzieren und exklusiv sein. Erstens stellen weder Mädchen noch Jungen eine homogene Adressat*innengruppe dar, sodass Interventionen, die 2 Versionen nach Geschlecht konzipieren, Unterschiede innerhalb von Geschlechtergruppen in unangemessener Weise homogenisieren könnten. Zweitens könnten bei der Erstellung einer männlichen und einer weiblichen Version stereotype binäre Geschlechtszuschreibungen vermittelt werden. Drittens bleibt die geschlechtliche Vielfalt unter Umständen unberücksichtigt, wodurch all jene nicht angesprochen werden, die sich nicht als (ausschließlich) männlich oder weiblich definieren können oder wollen (z. B. nichtbinäre Jugendliche).

Die Herausforderung bei der Forschung zu Geschlecht in der Gesundheitsförderung und Prävention sowie bei der Konzeption wirkungsvoller Interventionen besteht also darin, weder binäre Geschlechterzuschreibungen zu reproduzieren noch Geschlecht als Ungleichheits- und Analysekategorie aufzugeben.

Aufgrund der Potenziale von VR für gendersensible Gestaltung sollte nicht trotz, sondern gerade wegen dieser Herausforderung untersucht werden, welche Sichtweisen und Hinweise auf eine zugeschnittene, aber gleichzeitig genügend offene gendersensible Gestaltung innerhalb der Adressat*innengruppe bestehen. Dabei kann Geschlecht in seiner Komplexität ein erster Schritt sein, um im Sinne eines intersektionalen Verständnisses (s. z. B. Merz et al. für die Potenziale von Intersektionalität in Public Health [46]) weitere für die Adressat*innengruppe relevante Differenzkategorien (wie z. B. Bildung, Behinderung oder Betroffenheit von Rassismuserfahrungen) und ihre Überschneidungen zu berücksichtigen.

## Schlussfolgerungen für die Alkoholprävention

Die bisherigen Erfahrungen des Einsatzes von VR-basierten Alkoholpräventionsprogrammen an Schulen in Australien haben gezeigt, dass das Interesse von Schüler*innen an der Nutzung von VR groß ist [33]. Auch die Ergebnisse von Fokusgruppen mit Schüler*innen einer dänischen Internatsschule deuten darauf hin, dass die Schüler*innen die VR-Methode als attraktiver und innovativer bewerteten als herkömmliche Drogenpräventionsprogramme, mit denen sie Erfahrung gemacht haben [38]. Selbst wenn die Wirksamkeit der virtuellen Simulation zur Alkoholprävention auf die Steigerung der Kompetenz von Jugendlichen im Umgang mit Alkohol noch nicht nachgewiesen ist, lässt sich zumindest die Steigerung des Interesses von Schüler*innen an der Teilnahme an Alkoholpräventionsmaßnahmen bereits als Gewinn betrachten. In diesem Sinne könnten VR-Simulationen über den Umgang mit Alkohol in einer typischen Partysituation als Türöffner genutzt werden, um im Rahmen eines umfassenderen Präventionsprogramms mit anderen evidenzgestützten Maßnahmen kombiniert zu werden. Hier würde sich insbesondere die Kombination mit dem „Soziale-Normen-Ansatz“ anbieten, zumal Programme zur Steigerung der sozialen Kompetenz und Resilienz gegenüber Gruppendruck grundsätzlich mit Botschaften über soziale Normen verbunden werden sollten. Dies kann gesteigerten Normerwartungen bezüglich Alkoholkonsum entgegenwirken [47, 48].

Vorteil einer Anwendung virtueller Simulationsspiele in der schulischen Alkoholprävention ist neben den oben genannten Aspekten auch, dass die Nutzung durch den Download einer Smartphone-App in Kombination mit kostengünstigen VR-Brillen aus Pappe oder Kunststoff ohne aufwendige Investitionen in eine technische Ausstattung möglich ist. Schulen stehen häufig nur begrenzte Mittel zur Verfügung. Ebenso benötigen Pädagog*innen keine besondere Schulung, wie es z. B. für den sinnvollen Einsatz von Rollenspielen im Unterricht notwendig wäre. Eine Anleitung für Lehrkräfte, wie sie in Dänemark vom *VR-FestLab-*Forschungsteam entwickelt wurde (*VR FestLab* – sdu.dk), reicht aus, um die Anwendung der App im Unterricht anzuleiten. Darüber hinaus gibt die Anleitung Anregungen, wie die in der Simulation erlebten Erfahrungen sinnvoll in ein didaktisches Unterrichtskonzept zum Thema Kompetenz im Umgang mit Alkohol und Gruppendruckerfahrungen eingebettet werden können. An der Charité – Universitätsmedizin Berlin wird derzeit eine deutschsprachige Version der dänischen App *VR FestLab* namens *Virtual LimitLab* mit Jugendlichen getestet.

Die Grenzen der Methode bestehen darin, dass bei einer einmaligen Nutzung, z. B. im Rahmen von Projekttagen oder einer Unterrichtseinheit, nicht von einer nachhaltigen Wirkung ausgegangen werden kann, wie es auch in der randomisierten Studie bestätigt wurde [39]. Grundsätzlich ist die VR-Simulation für wiederholtes Spielen mit unterschiedlichen Spielverläufen im Sinne eines Kompetenztrainings hin zu einem erweiterten Handlungsrepertoire im Umgang mit sozialem Druck ausgelegt. Ob Jugendliche das Simulationsspiel jedoch außerhalb einer angeleiteten schulischen Alkoholprävention auch in ihrer Freizeit nutzen, ist noch nicht erwiesen. Die bisherigen Forschungsergebnisse [38] deuten darauf hin, dass Schüler*innen das Angebot einer VR-Alkoholprävention zwar sehr positiv bewerten, jedoch nur ca. die Hälfte der Befragten das Simulationsspiel auch Freund*innen empfehlen würde. Dennoch gab die überwiegende Mehrzahl an, das Spiel gerne weiter auszuprobieren, sodass in einem gewissen Umfang auch von einer Nutzung in der Freizeit auszugehen ist [38]. Dies könnte den Präventionseffekt erhöhen und auch zu einer weiteren Verbreitung innerhalb von Freundesgruppen beitragen. Grundsätzlich ist eine virtuelle Simulation zwar vermutlich geeignet, die Kompetenz von Jugendlichen im Umgang mit Alkohol zu stärken, aber der Ansatz bezieht sich in erster Linie auf die individuellen Ressourcen sowie das Verhalten von Schüler*innen und lässt die gesundheitsfördernde Veränderung der Lebensumwelt von Jugendlichen hingegen außen vor. Daher ist bei der Anwendung von Programmen zur Kompetenzerweiterung eine Begleitung mit umweltverändernden Maßnahmen in der Schule und im Umfeld der Schüler*innen erforderlich. Dies schließt ein Verbot jeglicher Alkoholwerbung im Umfeld von Schulen sowie eine mit Beteiligung der Schüler*innen konsentierte Alkoholpolitik der Schule ein. Nur dann können verhaltenspräventive und kompetenzorientierte Maßnahmen der schulischen Alkoholprävention erfolgreich sein.

## Fazit

Im Gegensatz zu geschriebenem Text, Fotografie oder Video ermöglicht VR eine immersive Erfahrung sowie eine Interaktion in Echtzeit. Außerdem kann mit VR in sehr wirkungsvoller Weise eine Geschichte erzählt und dargestellt werden, welche die Nutzenden auch emotional anspricht. Besonders wirksam sind Präventionsbotschaften, die auf die Rezipient*innen zugeschnitten sind, diese interaktiv einbeziehen und Kompetenzen im Umgang mit Alkohol und Gruppendruck vermitteln. Daher hat das Medium großes Potenzial im Bereich der Prävention und Gesundheitsförderung. Für die schulische Alkoholprävention mittels VR gibt es nun auch erste Erfolg versprechende Ansätze. Auch wenn es bisher nur wenige erprobte Anwendungen in Australien und Dänemark gibt, sollte das Potenzial für die schulische Alkoholprävention und auch dessen gendersensible Gestaltung weiter untersucht werden.
